# Self-Directed Walk With Ease Workplace Wellness Program — Montana, 2015–2017

**DOI:** 10.15585/mmwr.mm6746a3

**Published:** 2018-11-23

**Authors:** Robin P. Silverstein, Melissa VanderVos, Heather Welch, Alex Long, Charlotte D. Kaboré, Jennifer M. Hootman

**Affiliations:** ^1^Chronic Disease Prevention and Health Promotion Bureau, Montana Department of Public Health and Human Services; ^2^National Center for Chronic Disease Prevention and Health Promotion, CDC.

Arthritis occurs in 27% of adults in Montana, among whom 50% have activity limitations, 16% have social participation restrictions, and 23% have severe joint pain attributable to arthritis (*1*). Physical activity is beneficial in managing arthritis symptoms and in preventing other chronic diseases (*2*). Walk With Ease is a 6-week evidence-based physical activity program recommended by CDC to increase physical activity and help improve arthritis symptoms (*3*). In 2015, Walk With Ease was added to an ongoing workplace wellness program for Montana state employees; the results for five outcomes (minutes spent walking, engaging in other physical activity [including swimming, bicycling, other aerobic equipment use, and other aerobic exercise], stretching, pain, and fatigue) were analyzed by the Montana Department of Public Health and Human Services and CDC. Outcomes at baseline (pretest), 6 weeks after the program (posttest), and 6 months later (follow-up) were analyzed by self-reported arthritis status at the time the participant enrolled in the program. Significant increases (p<0.05) in the mean number of minutes spent per week walking and engaging in other physical activity were observed among participants with and without arthritis at the 6-week posttest. Time spent stretching did not change significantly at posttest for either group. Mean pain levels among participants without arthritis increased significantly both at the 6-week posttest and 6-month follow-up; however, pain and fatigue decreased significantly at posttest and follow-up for participants with or without arthritis who began the program with moderate or severe pain and fatigue levels. The data from these analyses suggest that, as a component of a workplace wellness program, self-directed Walk With Ease might be effective in increasing physical activity not only among adults with arthritis, but also among persons without arthritis.

Walk With Ease is a graduated walking program that can be delivered in an instructor-led group setting or through a self-directed workbook. Self-directed Walk With Ease participants communicate with a trainer by e-mail and walk on their own, reporting activity weekly. The Montana Health Care and Benefits Division has included self-directed Walk With Ease since 2015 in the established state employee wellness program, offering a health insurance premium discount as a financial incentive to participate in and complete the program.

Identical assessment surveys were conducted at the beginning of the program (at which time participants self-reported whether they had arthritis), immediately after the 6-week program, and at a 6-month follow-up. The surveys included questions on time spent engaged in six physical activities as well as levels of pain and fatigue, ranked on a scale of 0–10. The six physical activity questions asked how much time during the past week participants performed 1) stretching or strengthening exercises, 2) walking for exercise, 3) swimming or aquatic exercise, 4) bicycling (including using a stationary bike), 5) exercise using aerobic equipment other than a stationary bike, and 6) other aerobic exercise. Responses for the amount of time that participants engaged in physical activity, including walking and stretching, per week were coded as 0 (none), 1 (<30 minutes), 2 (30–60 minutes), 3 (61–180 minutes), or 4 (>180 minutes); these were converted to minutes (0, 15, 45, 120, and 240), respectively, for the analysis, using the median of each range and four hours for the highest category. Time spent engaged in other physical activity was obtained from the total number of minutes spent swimming, bicycling, using other aerobic equipment, and engaging in other aerobic exercise. 

To ascertain pain and fatigue, participants were asked how much pain or fatigue they experienced during the past week, on a scale from 0 (no pain or fatigue) to 10 (severe pain or fatigue), and were grouped into mild (1–3), moderate (4–6), and severe (7–10) categories for analysis. Data were analyzed for the period 2015–2017. Pretest, 6-week posttest, and 6-month follow-up data were compared using paired t-tests, with statistical significance defined as p<0.05.

The number of participants increased from 105 in 2015 to 1,343 in 2016, and to 1,622 in 2017. The majority of the 3,070 total participants during 2015–2017 were aged >45 years (75%), white (94%), women (72%), college graduates (63%), and with no disability (90%) or arthritis (76%) (Table 1). Among those participating in the program, 2,598 (85%) completed it by submitting weekly time spent engaged in physical activity during at least 4 of 6 weeks. Overall, 1,936 (63%) and 934 (30%) participants completed the 6-week posttest or 6-month follow-up survey, respectively. Among 743 (24%) persons with arthritis who started the program, 496 (67%) completed the 6-week posttest, and 279 (38%) provided 6-month follow-up data. Of the 2,327 (76%) persons without arthritis who started the program, the 6-week posttest and 6-month follow up was completed by 1,440 (62%) and 655 (28%) participants, respectively. (Table 1).

**TABLE 1:** Characteristics of state employee Walk With Ease participants (N = 3,070) and percentage completing pretest and 6-week posttest or 6-month follow-up surveys, by arthritis status — Montana, 2015–2017

Characteristic (no. with available information*)	Participants			Participants completing pretest and 6-week posttest		Participants completing pretest and 6-month follow-up	
	No. (%)	With arthritis %	No arthritis %	With arthritis %	No arthritis %	With arthritis %	No arthritis %
**Total**	**3,070 (100)**	**24.2**	**75.8**	**66.8**	**61.9**	**37.6**	**28.1**
**Age group (yrs) (2,970)**							
21–34	307 (10)	6.5	93.5	75.0	57.5	45.0	17.8
35–44	452 (15)	11.1	88.9	50.0	54.0	20.0	18.7
45–54	790 (27)	19.6	80.4	64.5	63.3	37.4	32.3
55–64	1,099 (37)	33.5	66.5	66.3	67.4	39.9	34.9
≥65	322 (11)	42.2	57.8	75.0	71.0	34.6	31.7
**Sex (2,949)**							
Women	2,113 (72)	26.7	73.3	64.4	60.4	39.5	30.0
Men	836 (28)	19.3	80.7	73.9	67.0	31.7	24.4
**Education (2,923)**							
High school graduate	342 (12)	28.7	71.3	66.3	65.2	34.7	33.6
Some college	761 (26)	28.5	71.5	66.8	63.6	33.2	28.7
College graduate	1,278 (44)	21.8	78.2	66.7	63.7	40.1	29.3
Graduate school	542 (19)	24.9	75.1	65.2	61.7	41.5	25.8
**Race (2,994)**							
American Indian	78 (3)	38.5	61.5	60.0	58.3	40.0	18.8
White	2,812 (94)	25.1	74.9	67.4	63.6	37.9	29.0
Other race	104 (3)	14.4	85.6	46.7	51.7	33.3	29.2
**Disability status (2,767)**							
Disability^†^	269 (10)	48.7	51.3	58.8	65.9	39.7	29.7
No disability	2,498 (90)	21.3	78.7	72.2	65.0	37.3	29.2
**Baseline pain (2,909)**							
None (0)	817 (28)	6.4	93.6	71.2	64.1	42.3	30.6
Mild (1–3)	1,282 (44)	21.9	78.1	75.4	68.3	40.2	31.8
Moderate (4–6)	617 (21)	44.7	55.3	64.1	63.6	38.8	26.1
Severe (7–10)	193 (7)	61.7	38.3	55.5	56.8	34.5	27.0
**Baseline fatigue (2,913)**							
None (0)	716 (25)	15.1	84.9	81.5	67.4	44.4	33.4
Mild (1–3)	1,143 (39)	21.4	78.6	67.3	67.4	42.9	31.3
Moderate (4–6)	683 (23)	31.3	68.7	67.8	62.9	35.5	26.2
Severe (7–10)	371 (13)	43.7	56.3	58.6	59.3	34.6	28.2

* Participants without available information were as follows: age group, 100 (3.3%); sex, 121 (3.9%); education, 141 (4.6%), and less than high school graduate excluded for six (0.2%); race, 76 (2.5%); disability status, 303 (9.9%); baseline pain, 161 (5.2%); baseline fatigue, 157 (5.1%).

^†^ Based on self-reported hearing, seeing, walking, climbing stairs, or cognitive disability.

Among participants with and without arthritis, the mean number of minutes per week spent walking and engaged in other physical activity significantly increased at posttest. Overall, 73% of persons who walked <30 minutes per week at pretest increased to >60 minutes at posttest. Similarly, the majority of those starting at <30 minutes increased to >30 minutes at the 6-month follow-up (Figure). From a baseline of no walking, 97% were walking at 6 weeks, with 65% walking >60 minutes per week; 87% continued walking at the 6-month follow-up, with 35% walking >60 minutes per week. Among all participants who began the program walking 1–3 hours and >3 hours per week, 89% and 72%, respectively, maintained or increased their time spent walking at posttest. Mean number of minutes spent stretching did not change significantly at posttest. Mean walking and other physical activity did not change significantly at 6-month follow-up, except for a significant increase in walking when considering all participants. Stretching increased significantly at 6 months only for those with no arthritis (Table 2).

**FIGURE:**
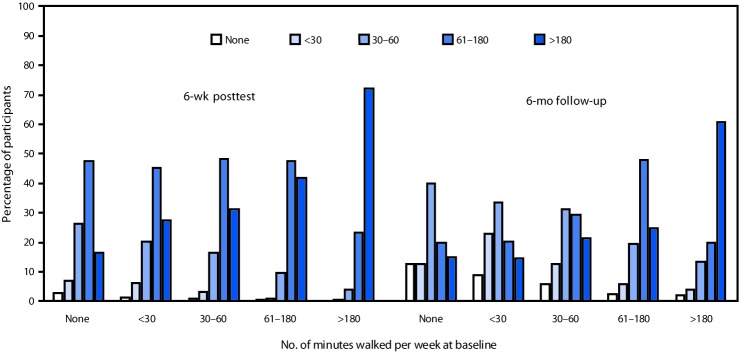
Percentage of participants, grouped by baseline walking, by number of minutes spent walking per week at the 6-week posttest and 6-month follow-up, among state employees participating in a self-directed Walk With Ease program — Montana, 2015–2017

**TABLE 2:** Mean changes in minutes walked and pain and fatigue scores* for state employee Walk With Ease participants who completed pretest and 6-week posttest or 6-month follow-up surveys, by arthritis status — Montana, 2015–2017

Variable	With arthritis			No arthritis			Overall		
	Pretest	Posttest/Follow-up	Change	Pretest	Posttest/Follow-up	Change	Pretest	Posttest/Follow-up	Change
**6-week posttest**									
**Physical activity (minutes per week)**									
Walking	107.5	156.5	49.1^†^	119.7	163.6	43.8^†^	116.6	161.8	45.2^†^
Other physical activity^§^	50.3	64.6	14.3^†^	69.8	88.1	18.3^†^	64.9	82.2	17.3^†^
Stretching	51.7	57.2	5.5	55.2	56.5	1.3	54.3	56.7	2.3
**Baseline pain level (scale of 0–10)**									
all (0–10)	3.77	3.74	-0.03	1.87	2.12	0.25^†^	2.35	2.53	0.18^†^
zero (0)	0.00	1.41	1.41^†^	0.00	1.13	1.13^†^	0.00	1.15	1.15^†^
mild (1–3)	2.17	3.00	0.83^†^	1.92	2.19	0.27^†^	1.98	2.38	0.40^†^
moderate (4–6)	4.97	4.36	-0.60^†^	4.82	3.66	-1.16^†^	4.89	3.98	-0.91^†^
severe (7–10)	7.80	5.74	-2.06^†^	7.57	4.48	-3.10^†^	7.71	5.25	-2.46^†^
**Baseline fatigue level (scale of 0–10)**									
all (0–10)	3.61	3.36	-0.25^†^	2.50	2.33	-0.18^†^	2.79	2.59	-0.20^†^
zero (0)	0.00	1.52	1.52^†^	0.00	0.93	0.93^†^	0.00	1.04	1.04^†^
mild (1–3)	1.95	2.68	0.74^†^	1.98	2.20	0.22^†^	1.97	2.30	0.33^†^
moderate (4–6)	4.90	3.85	-1.05^†^	4.74	3.49	-1.25^†^	4.79	3.61	-1.18^†^
severe (7–10)	7.88	5.48	-2.40^†^	8.02	4.81	-3.21^†^	7.96	5.10	-2.86^†^
**6-month follow-up**									
**Physical activity (minutes per week)**									
Walking	106.4	116.9	10.5	126.2	132.0	5.7	120.3	127.5	7.1^†^
Other physical activity^§^	53.0	62.3	9.3	68.0	74.6	6.7	63.6	71.0	7.4
Stretching	54.7	59.8	5.1	56.0	62.9	6.9^†^	55.6	62.0	6.4^†^
**Baseline pain level (scale of 0–10)**									
all (0–10)	3.90	3.82	-0.08	1.79	2.24	0.45^†^	2.42	2.72	0.29^†^
zero (0)	0.00	1.64	1.64^†^	0.00	1.52	1.52^†^	0.00	1.53	1.53^†^
mild (1–3)	2.26	3.29	1.04^†^	1.93	2.39	0.46^†^	2.01	2.63	0.61^†^
moderate (4–6)	4.93	4.06	-0.87^†^	4.73	3.26	-1.47^†^	4.84	3.69	-1.14^†^
severe (7–10)	7.85	5.85	-2.00^†^	7.40	3.85	-3.55^†^	7.70	5.20	-2.51^†^
**Baseline fatigue level (scale of 0–10)**									
all (0–10)	3.56	3.69	0.13	2.39	2.66	0.27^†^	2.74	2.97	0.23^†^
zero (0)	0.00	2.15	2.15^†^	0.00	1.22	1.22^†^	0.00	1.39	1.39^†^
mild (1–3)	1.89	3.03	1.14^†^	1.89	2.66	0.77^†^	1.89	2.76	0.87^†^
moderate (4–6)	4.97	4.51	-0.46^†^	4.76	3.93	-0.82^†^	4.84	4.16	-0.68^†^
severe (7–10)	7.82	5.14	-2.68^†^	8.02	4.95	-3.07^†^	7.92	5.04	-2.88^†^

* Increasing pain and fatigue were ranked on a 0–10 scale. All (0–10) is the nonstratified mean value. Mean values within subcategories of baseline pain and fatigue were defined as follows: zero = 0, mild = 1–3, moderate = 4–6, and severe = 7–10.

^†^ p-value <0.05

^§^ Total minutes spent swimming, bicycling, using exercise equipment, or engaged in other aerobic exercise.

Among participants without arthritis, mean reported pain level increased significantly at 6 weeks and 6 months. Mean level of fatigue decreased significantly for both those with arthritis and no arthritis at 6 weeks, but increased significantly for those without arthritis at 6 months. 

Among participants who started with moderate or severe pain and fatigue levels, mean pain and fatigue decreased significantly at posttest and 6-month follow-up among participants with and without arthritis (Table 2), whereas among participants starting the program with low levels of pain and fatigue, small but significant increases in mean pain and fatigue at the 6-week posttest and 6-month follow-up occurred for participants with and without arthritis (Table 2).

## Discussion

Participants with and without self-reported arthritis in the self-directed Walk With Ease program in Montana experienced significant increases at the 6-week posttest in the mean number of minutes per week spent walking. Increases in the number of minutes spent walking each week were consistent with findings from other evaluations of Walk With Ease, which showed improved performance measures, self-reported outcomes, and well-being among persons with self-reported arthritis (*3*,*4*). The majority of participants who reported little (<30 minutes a week) or no walking at the start were walking >60 minutes at the 6-week posttest and >30 minutes at the 6-month follow-up. Most walkers who started at other levels (>30 minutes a week) also maintained or increased their walking levels. The advances in walking at 6 weeks, however, were diminished at 6 months, suggesting that additional efforts might be needed to sustain program gains.

A notable finding of the analysis of the Walk With Ease program was the increase in time spent walking among persons who were not walking at all at the start of the intervention. The Walk With Ease program has the potential to offer a substantial change for adults with a sedentary lifestyle, who could benefit from public health interventions that encourage and promote a physically active lifestyle.

Lack of significant improvements in pain overall differed from previous findings, which showed that the walking programs reduced pain (*4*–*6*). The significantly increased pain levels among those with no arthritis were partially the result of regression toward the mean, with 48% of the participants starting with mild pain levels and 34% starting with zero pain. Short-term variability in pain scores has been documented, with more than 50% of veterans with chronic pain showing a range of more than two points on the 0–10 numeric rating scale in a given month (*7*). The pattern of worsening pain for those with low starting pain and improvements for those with moderate to severe pain mirrors that found in other research, which documented increased pain among persons starting with low pain and improvement in pain for those starting with moderate to severe pain (*8*,*9*). An improved statistical measure to examine the pain scale when applied to healthier populations with many participants starting with no pain might be needed (*10*).

The findings in this report are subject to at least five limitations. First, Walk With Ease outcomes are based on self-reporting of time engaged in physical activity and pain and fatigue symptoms, which are subject to recall and social desirability biases. Second, pain levels might include a short-term increase of symptoms from new exercise, with later improvement; information on location of pain in the surveys might have improved this. Third, there was no control group to examine whether changes in pain and fatigue resulted from regression to the mean or from the intervention. Fourth, the 6-month follow-up had a low overall completion rate (30%), which could introduce reporting bias if outcomes influenced reporting and if persons who maintain the benefits long-term are more likely to respond. Finally, arthritis was self-reported and not confirmed by medical record review or clinical evaluation, which could have resulted in misclassification of arthritis status.

Self-directed Walk With Ease increased physical activity among adults with and without self-reported arthritis, particularly among persons with low levels of activity at baseline, and decreased pain and fatigue among those reporting moderate or severe pain and fatigue at baseline. Walk With Ease can succeed as a workplace wellness program for employees with and without arthritis, moving persons from no activity to some activity and promoting the general health benefits from improved physical activity. Public health professionals might consider Walk With Ease and similar fitness programs in collaboration with employers to improve worker health.

**Summary**

**What is already known about this topic?**

The Walk With Ease exercise program can improve arthritis symptoms and increase physical activity.

**What is added by this report?**

Among Montana state workers with and without self-reported arthritis who participated in self-directed Walk With Ease, walking levels increased significantly. Among participants not walking for exercise at the start of the program, 97% were walking at 6 weeks and 87% at 6 months. Pain and fatigue decreased among those with moderate or severe pain or fatigue at baseline.

**What are the implications for public health practice?**

Walk With Ease fits in workplace wellness programs, increases physical activity, and might help prevent future chronic diseases. Public health professionals can promote physical activity programs in collaboration with employers to improve worker health.
